# Oral Lichenoid Lesion Manifesting as Desquamative Gingivitis: Unlikely Association? Case Report

**DOI:** 10.2174/1745017901814010679

**Published:** 2018-09-28

**Authors:** Lívia Maria Lopes de Oliveira, Luiz Henrique Carvalho Batista, Alexandrino Pereira dos Santos Neto, Luciano Barreto Silva, Renata Cimões, Jair Carneiro Leão, Maria Leticia Cintra, Camila Maria Béder Ribeiro

**Affiliations:** 1 Department of Prosthesis and maxillofacial surgery, Faculty of Dentistry, Federal University of Pernambuco Recife, Brazil; 2 Department of Periodontology, Dental School, Cesmac University Center, Maceió, Brazil; 3 Medical Sciences College, University of Campinas – FCM UNICAMP, Campinas, Brazil; 4 Department of Pathology, Faculty of Dentistry, Cesmac University Center, Cônego Machado, 918, Farol CEP: 57051-160, Maceió, Alagoas, Brazil

**Keywords:** Dental amalgam, Gingivitis diseases, Delayed hypersensitivity, Lichenoid lesions, Oral lichen planus, Heterogeneous clinical manifestations

## Abstract

**Introduction:**

The aim of this report is to present a clinical case of oral lichenoid lesions associated with amalgam restorations with the presence of desquamative gingivitis for a nine months follow up period.

**Case Report:**

The histopathologic characteristics and direct immunofluorescence were compatible with Oral Lichenoid Lesion (LLO). Diagnosis was based on a synthesis of all available information, including medical history, clinical examination, histopathology and the results of specific tests, such as the patch test, which confirmed allergy to thimerosal, an organic compound of mercury.

**Discussion:**

The replacement of amalgam restorations has brought improvements to the instrument, as evidenced by the disappearance of desquamative gingivitis, aspect erythematosus and erosive lesions. The fading does not complete the same, however, indicates the need to continue has been under continuous observation, the patient, having in view the possibility of the existence of an underlying lichen planus.

## 1 INTRODUCTION

Oral Lichenoid Lesions, or Reactions (OLLs/OLRs), have been defined as clinical and histopathological features of Oral Lichen Planus (OLP) with identifiable etiology. Lichenoid lesions have also been associated with Dental Amalgam (OLL-DA), representing type IV hypersensitivity reaction, often referred to as delayed hypersensitivity, since it generally develops after a relatively long-time period, in this case, from months to years. OLLs generally show heterogeneous clinical manifestations ranging from white asymptomatic plaque lesions to atrophic, symptomatic reticular lesions, erosive lesions [1, 2] or even scaly lesions [3].

When the clinical aspect described occurs in marginal and/or inserted gingiva, the clinical term Desquamative Gingivitis (DG) is used. The latter is not considered a definite pathology, but rather a common sign of several pathologies, encompassing a broad clinical spectrum of mucocutaneous diseases ranging from vesiculobullous immune disorders to allergic reactions to a number of chemical products or allergens [4, 5].

The possible correlation between the presence of DG associated with autoimmune diseases and the worsening of periodontal parameters has been discussed in the literature, since both conditions can share immuno-inflammatory mechanisms in their pathogenesis. DG may indirectly worsen the accumulation of plaque in the affected sites, since pain and bleeding may inhibit the patient’s oral hygiene. Microorganisms from the oral biofilm trigger, *via* immunological mechanisms from innate and adaptive immunities, an immune response which activates inflammatory mediators such as cytosines and Metalloproteinases (MMPs) [6, 7]. Their serum levels also increased in the presence of oral lichen planus [8], commonly associated with DG [9-11].

Diagnosis must be based on a synthesis of all available information, including medical history, clinical examination and histopathology as well as the results of specific tests, such as the patch test [12-18] The replacement of amalgam fillings by other restorative materials is usually enough to improve or solve the OLL-DA [16, 19, 20]. It is common knowledge that basic periodontal therapy and plaque control promote serum reduction of inflammatory immune mediators [21, 22], and may act synergistically with the removal of restorative materials in order to treat gingival lesions.

There have been few reports associating desquamative gingivitis and OLL-DA available in the current literature. Therefore, the aim of this study is to report a clinical case of idiopathic OLL caused by an allergic reaction to thimerosal associated with desquamative gingivitis in a patient with periodontal disease.

## 2 CASE REPORT

**Fig. (1):**
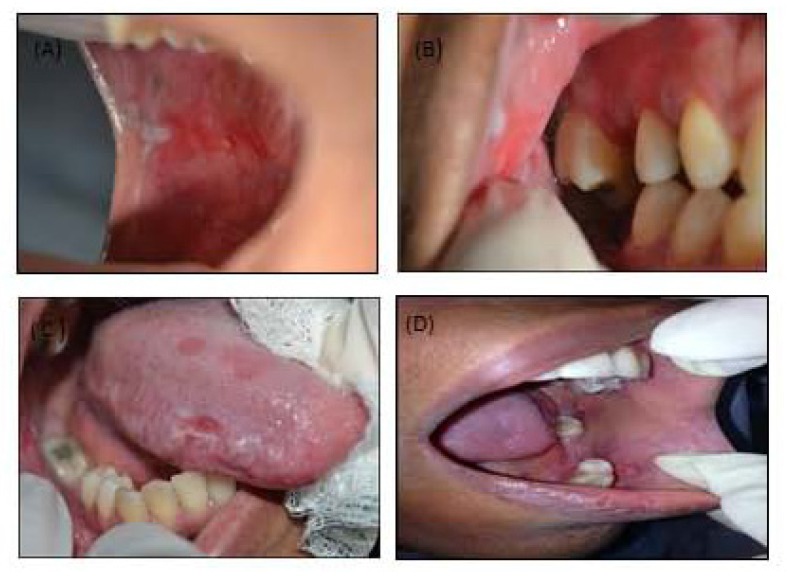
Initial clinical aspects. **A**) Lesion on the buccal mucosa, multiple reticular and leucoplast lesions, slightly erythematous with atrophic areas and ulcerated surfaces, are observed. **B**) Desquamative and erythematous appearance of the inserted gingiva, and ulcerated region in the buccal mucosa. **C**) Erythematous area with ulcer in the center and variation of normality, geographical language. **D**) Absence of lesions contralaterally.

A 33-year-old woman sought dental assistance and presented multiple unilateral lesions distributed throughout the oral mucosa, retro commissural region, inserted gingiva lateral borders of the tongue on the right side (Figs. **1A-D**) and *desquamative* gingivitis located in the inserted gingiva. The asymptomatic lesions showed an atrophic leuco-erythroblastic reticular aspect, with an ulcerated surface. Teeth 17, 15, 14 and 48 had extensive amalgam restorations two years prior, in close contact with the injured areas.

During the anamnesis, she reported having been submitted to periodontal therapy. The main complaint motivating her search for professional assistance was halitosis associated with spontaneous gingival bleeding. Periodontal examination was carried out with a periodontal probe (PCPUNC15, Hu-Friedy, Chicago, IL, USA) and the measure of probing depth was registered, as well as the presence of bleeding during the probing step (MÜHLEMANN; SON, 1971) and the plaque index (O’LARY; DRAKE; NAYLOR, 1972). The gingival depth ranged from 1 to 7 mm; however, in the region affected by the gingival desquamation, the depths ranged from 2 to 6 mm. The bleeding index during probing was 91.3%, and the plaque index was 74%. With such results, the patient was diagnosed with generalized periodontitis. The patient’s medical history did not reveal any systemic alterations, such as hypertension, diabetes or any autoimmune diseases, nor did she mention the use of any medication. Blood analysis did not reveal any alterations.

**Fig. (2):**
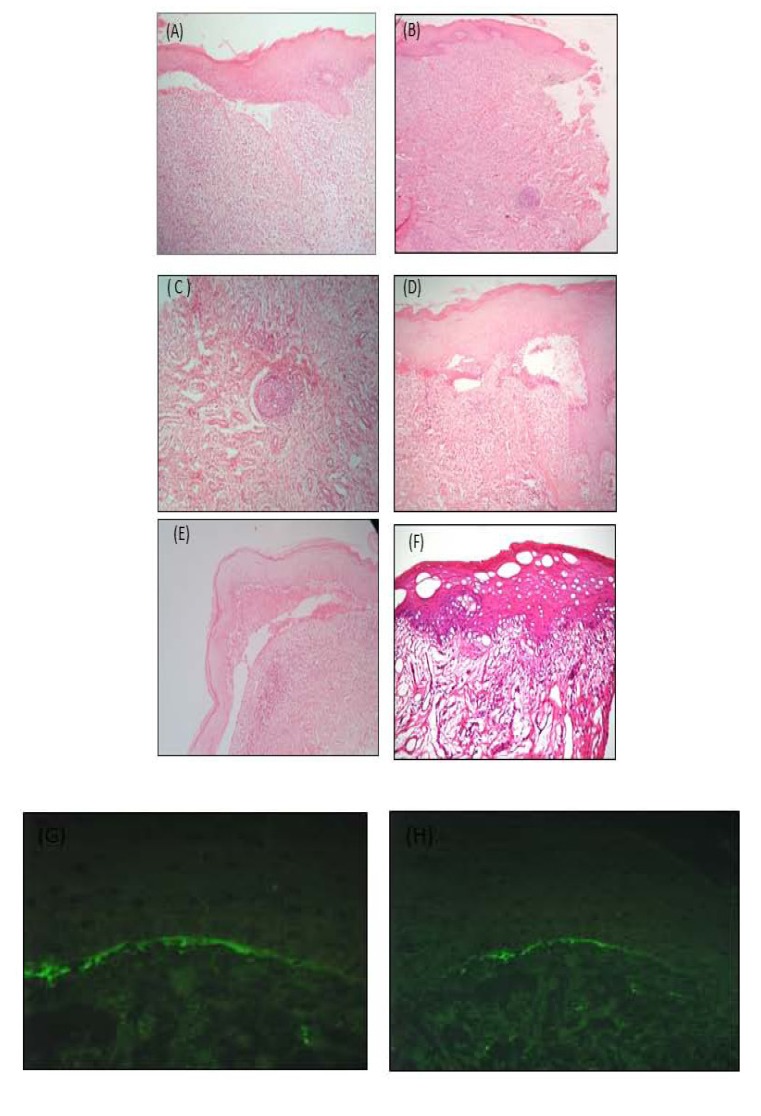
Histolopathological and direct immunofluorescence aspects of buccal mucosa and gingiva lesions. **A**) Histological cut of the buccal mucosa in HE staining (X100). **B**) Buccal mucosa fragment showing subepithelial lymphoplasmacytic inflammatory infiltrate and lymphoid follicle formation HEx100. **C**) Lymphoid follicle HEx40. **D**) Stratified keratinized squamous epithelium, chronically inflamed conjunctiva. **E**) Subepithelial cleft in the gingival region. **F**) Cornified hyperplasia and metaplasia of the stratified squamous epithelium, sparse apoptotic cells, pigmentary incontinence and moderate superficial interstitial lymphohistiocytic inflammatory infiltrate. (*) Artifacts: Retraction of both epithelial cells and collagen, leaving spaces. **G**) Focal granular deposit of IgM 2+ (on a scale of 1+ to 3+) in the basement membrane zone of the epidermis. **H**) The same in lower increase.

A perilesional biopsy was then carried out using hematoxylin-eosin staining. The oral mucosa fragment was covered by focal acantholytic, atrophic and parakeratinized squamous epithelium. The connective tissue showed chronic subepithelial and deep inflammatory infiltrate, predominantly composed of lymphocytes and plasmocytes, with the formation of lymphoid follicles and subepithelial cleft, compatible with oral lichenoid reaction (Figs. **2A**-**C**). The histological section of the gingiva also revealed chronically inflamed connective tissue and subepithelial cleft (Figs. **2D**-**F**) A provisional diagnosis of OLL-DA was initially taken. The patch test reading confirmed allergy to thimerosal, a compound of mercury metal. Therefore, all the metallic restorations were removed in one single appointment. Afterwards, basic periodontal therapy was accomplished in four consecutive weekly appointments, with scaling and root debridement, structured plaque control, dental brushing and interdental cleaning instructions.

**Fig. (3):**
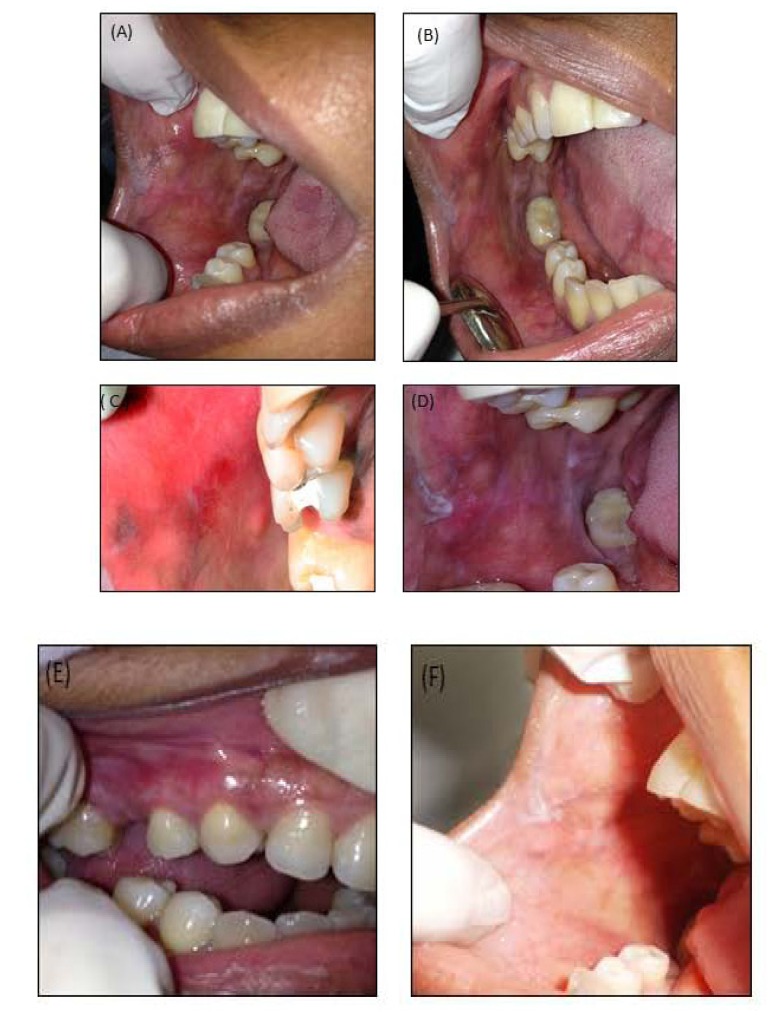
Clinical aspects after removal of amalgam restorations. **A**) Following three months, buccal mucosa: there was a reduction of the erythroplasia area and persistence of the leucoplast plaque associated with element 15, which presented failure of the temporary restoration. **B**) Following six months: Detail of the lateral margin of the tongue, disappearance of ulcerated and erythematous area; Observation of residual leucoplast area in the buccal mucosa, and retro commissural region. **C**) Detail of the residual erythematous area. **D**) Following six months, there was an improvement in the leuco-erythroblastic appearance. However, development of more intense leucoplast striations around element 48 and development of an erythematous area adjacent to the leucoplast plaque in the retro commissural region, with ulceration in the center were observed. **E**) Following nine months: Remission of desquamative gingivitis was observed. **F**) Persistence of more concentrated leucoplast reticular lesions in the retro commissural region.

The follow-up period ranged from three (Figs. **3A** & **C**) to six (Figs. **3B** & **D**) months, during which time the desquamative gingivitis was reduced. However, the leucoplast lesions located on the retro commissural area and tongue were unchanged. A new perilesional biopsy for the direct immunofluorescence test was conducted in order to exclude other diagnostic hypotheses. The results in question showed non-specific focal granular deposits of IgM 2+ (on a scale of 1+ to 3+) in the basement membrane zone of the epidermis (Figs. **2G** & **H**). The findings were compatible with Lichenoid Stomatitis. After clinical-pathological correlation, the diagnosis of idiopathic oral lichenoid lesion was finally established. Nine months after the removal of all the amalgam restorations, remission of the desquamative gingivitis (Fig. **3E**) and disappearance of reddish-white plaques, as well as the ulcerated surface of the oral mucosa were observed. Nevertheless, the reticular leucoplast and retro commissural region lesions persisted (Fig. **3F**).

## 3 DISCUSSION

Lichenoid Lesions associated with Dental Amalgam (OLL-DA) represent a late hypersensitivity response to some components of metal alloys, particularly mercury [7]. Clinically and histologically, OLL may be indistinguishable from Oral Lichen Planus (OLP) and present underlying desquamative gingivitis [18].

OLLs caused by hypersensitivity to amalgam or its constituents typically have a clear anatomical relationship with dental restorations, and are usually unilateral and non-symmetric [2]. OLLs are detected in the buccal mucosa and lateral portion of the tongue and, less frequently, in the gingiva [1]. The clinical aspect of this case resembled the description often found and described in the current literature.

There is a difficulty in establishing the differential diagnosis between OLP and OLL-DA due to similarities between them, which often makes them clinically and histologically indistinguishable [15]. Mravak-Stipetić *et al*., [13], in a study correlating the clinical and pathological characteristics of OLP with OLL, found a coincidence of nearly 50% between histological findings and clinical diagnosis, concluding that histological characteristics themselves may not always be exclusive in the interpretation for diagnosis. The initial clinical diagnostic hypothesis was OLL-DA, in view of the location of the lesions and the reticular, atrophic and slightly erythematous aspect of the buccal mucosa and tongue [2, 20].

Histopathological examination revealed interstitial lymphohistiocytic inflammatory infiltrate in the subepithelial band, with fibrosis in some points and presence of lymphoid follicles. These characteristics are in agreement with those described in literature studies [23] concerning the histology of OLL-DA. Descaling gingivitis, described by histopathology, however, has made it difficult to establish a conclusive diagnosis, although the histopathological reports of the buccal mucosa and tongue confirm the OLL-DA hypothesis. Following the clinical flow chart proposed by Al-Abeedi *et al*., [24], all of the following were taken into account for the achievement of the diagnosis: the patient's clinical and medical history, proximity to the gingival lesion, extensive amalgam restorations, negative Nikolsky's signal, absence of other lesions on the skin and mucosa, absence of systemic signs and symptoms, non-existence of history of cardiovascular problems, use of medications, chronic character of the lesions.

Essentially, OLL-DA is the equivalent, in the oral environment, to allergic contact dermatitis. In most cases, the allergen is mercury, but occasionally some other alloy components may trigger a reaction [25]. In the case report presented in this paper, the patch test reading showed that the patient was allergic to thimerosal (a compound of organic mercury and thiosalicylate), implicated as the most common inducer of cutaneous reactions. Scalf *et al*., [26] showed a 25.5% prevalence of thimerosal allergy in patients with a history of lichenoid lesions. Despite the generalized use of dental amalgam as a dental material, reports of hypersensitivity cases to amalgam, or Lichenoid Lesion associated with Dental Amalgam (OLL-DA), are uncommon. However, studies have revealed hypersensitivity reactions due to contact with dental materials, such as amalgam, presenting an aspect of oral lichenoid lesion [19] similar to the test case.

Histopathological examination performed after the removal of the amalgam restorations showed the disappearance of the lymphoid follicle. Ribeiro and Larsson [23, 27] distinguished the frequent presence of this structure in histological findings of biopsies with diagnosis of OLL-DA. According to Larsson and Warfvinge [27], the structure of this lymphoid organ, similar to that of the secondary follicles, is induced in the peripheral tissue by an antigen locally responsible for the maintenance of an abnormal immune response. The removal of such antigens, in this case mercury in organic form present in amalgam restorations, would be responsible for its disappearance.

Three months following the replacement of the restorations, there was an improvement of the clinical aspect (Fig. **3A**) of the buccal mucosa and tongue. However, there was still the persistence of leucoplast plaque in the retro commissural area (Fig. **3A**) and a residual erythematous area (Fig. **3C**) related to tooth 15, whose provisional filling had failed. Montebugnoli *et al*., and Pawar *et al*., [15, 28] observed partial or complete remission of the lesions in the three-month period. The residual erythematous area, observed at three months on the buccal mucosa, probably persisted because there was contact between it and the dentin surface exposed by the failure of the provisional restoration. During this period, basic periodontal therapy was concluded, with reduction of periodontal indices related to gingival bleeding and plaque accumulation, according to the literature [22]. The persistence of the residual erythematous area, however, suggests that scaling and root debridement may have contributed to the improvement, but was not enough to solve DG.

In the six month clinical examination, clinical improvement and disappearance of desquamative gingivitis were observed. However, there were persistent reticular white lesions coincident with the occlusion line in the buccal mucosa and ulceration in the center of an erythematous area adjacent to the retro commissural area and leucoplast plaques on the lateral side of the tongue and retro commissural area (Fig. **3B** and **D**).

After nine months, although there were no indications of desquamative gingivitis (Fig. **3E**) or ulcerations, smoothed reticular lines in the buccal mucosa were observed (Fig. **3F**). It is speculated that in this case, the persistence of lesions in the other sites was due either to exclusively unilateral mastication, which promoted trauma and subsequent development of hyperkeratosis, or to scarification in the region of the oral lichenoid lesion, or to the persistence of an underlying lichen planus.

## CONCLUSION

The complete healing of the desquamative gingivitis, after this period, however, reinforced that it was related to the development of lichenoid lesions associated with dental amalgam, as a hypersensitive response to thimerosal. Although basic periodontal therapy may have contributed to the resolution of the DG, it was not sufficient, since the complete removal of dental amalgam restorations was necessary for the complete disappearance of the erythematous areas of the gingiva.

## References

[1] Lartitegui-Sebastián M.J., Martínez-Revilla B., Saiz-Garcia C., Eguizabal-Saracho S., Aguirre-Urizar J.M. (2012). Oral lichenoid lesions associated with amalgam restorations: A prospective pilot study addressing the adult population of the Basque Country.. Med. Oral Patol. Oral Cir. Bucal.

[2] McParland H., Warnakulasuriya S. (2012). Oral lichenoid contact lesions to mercury and dental amalgam: A review.. J. Biomed. Biotechnol..

[3] Rogers R.S., Sheridan P.J., Jordon R.E. (1976). Desquamative gingivitis: Clinical, histopathologic, and immunopathologic investigations.. Oral Surg. Oral Med. Oral Pathol..

[4] Lo Russo L., Gallo C., Pellegrino G., Lo Muzio L., Pizzo G., Campisi G., Di Fede O. (2014). Periodontal clinical and microbiological data in desquamative gingivitis patients.. Clin. Oral Investig..

[5] Monea M., Hănțoiu T., Stoica A., Vlad R., Sitaru A. (2017). The Influence of desquamative gingivitis on periodontal health.. J Interdisciplin Med.

[6] Björnfot Holmström S., Clark R., Zwicker S., Bureik D., Kvedaraite E., Bernasconi E., Nguyen Hoang A.T., Johannsen G., Marsland B.J., Boström E.A., Svensson M. (2017). Gingival tissue inflammation promotes increased matrix metalloproteinase-12 production by cd200r^low^ monocyte-derived cells in periodontitis.. J. Immunol..

[7] Cavalla F., Biguetti C.C., Garlet T.P., Trombone A.P.F., Garlet G.P., Bostanci N., Belibasakis G. (2018). Inflammatory pathways of bone resorption in periodontitis.. Pathogenesis of periodontal diseases..

[8] Ertugrul A.S., Dursun R., Dundar N., Avunduk M.C., Hakki S.S. (2013). MMP-1, MMP-9, and TIMP-1 levels in oral lichen planus patients with gingivitis or periodontitis.. Arch. Oral Biol..

[9] Lo Russo L., Fierro G., Guiglia R., Compilato D., Testa N.F., Lo Muzio L., Campisi G. (2009). Epidemiology of desquamative gingivitis: Evaluation of 125 patients and review of the literature.. Int. J. Dermatol..

[10] Maderal A.D., Lee Salisbury P., Jorizzo J.L. (2018). Desquamative gingivitis: Clinical findings and diseases.. J. Am. Acad. Dermatol..

[11] Leão J.C., Ingafou M., Khan A., Scully C., Porter S. (2008). Desquamative gingivitis: Retrospective analysis of disease associations of a large cohort.. Oral Dis..

[12] Suter V.G., Warnakulasuriya S. (2016). The role of patch testing in the management of oral lichenoid reactions.. J. Oral Pathol. Med..

[13] Mravak-Stipetić M., Lončar-Brzak B., Bakale-Hodak I., Sabol I., Seiwerth S., Majstorović M., Grce M. (2014). Clinicopathologic correlation of oral lichen planus and oral lichenoid lesions: A preliminary study.. Sci. World J..

[14] Rai R., Dinakar D., Kurian S.S., Bindoo Y.A. (2014). Investigation of contact allergy to dental materials by patch testing.. Indian Dermatol. Online J..

[15] Montebugnoli L., Venturi M., Gissi D.B., Cervellati F. (2012). Clinical and histologic healing of lichenoid oral lesions following amalgam removal: A prospective study.. Oral Surg. Oral Med. Oral Pathol. Oral Radiol..

[16] Mårell L., Tillberg A., Widman L., Bergdahl J., Berglund A. (2014). Regression of oral lichenoid lesions after replacement of dental restorations.. J. Oral Rehabil..

[17] Dudhia B.B., Dudhia S.B., Patel P.S., Jani Y.V. (2015). Oral lichen planus to oral lichenoid lesions: Evolution or revolution.. J. Oral Maxillofac. Pathol..

[18] Carbone M, Broccoletti R, Gambino A (2015). Clinical and histological features of gingival lesions: A 17-year retrospective analysis in a northern Italian population.. Med. Oral Patologia Oral Y Cirugia Buccal.

[19] Kamath V.V., Setlur K., Yerlagudda K. (2015). Oral lichenoid lesions: A review and update.. Indian J. Dermatol..

[20] van der Meij E.H., van der Waal I. (2003). Lack of clinicopathologic correlation in the diagnosis of oral lichen planus based on the presently available diagnostic criteria and suggestions for modifications.. J. Oral Pathol. Med..

[21] Mastromatteo-Alberga P., Escalona L.A., Correnti M. (2018). Cytokines and MMPs levels in gingival crevicular fluid from patients with chronic periodontitis before and after non-surgical periodontal therapy.. J Oral Res.

[22] Stone S.J., Heasman P.A., Staines K.S., McCracken G.I. (2015). The impact of structured plaque control for patients with gingival manifestations of oral lichen planus: A randomized controlled study.. J. Clin. Periodontol..

[23] Ribeiro C.M.B. T-helper-1 and 2 (Th1/2) responses polymorphisms in immunologically mediated diseases with oral manifestations: Oral lichen planus and oral lichenoid reaction by dental amalgam..

[24] Al-Abeedi F., Aldahish Y., Almotawa Z., Kujan O. (2015). The differential diagnosis of desquamative gingivitis: Review of the literature and clinical guide for dental undergraduates.. J. Int. Oral Health.

[25] Muris J., Kleverlaan C.J., Chen J., Thyssen J. (2018). Hypersensitivity to dental alloys. A Metal allergy.. Metal Allergy..

[26] Scalf L.A., Fowler J.F., Morgan K.W., Looney S.W. (2001). Dental metal allergy in patients with oral, cutaneous, and genital lichenoid reactions.. Am. J. Contact Dermat..

[27] Larsson A., Warfvinge G. (1998). Immunohistochemistry of ‘tertiary lymphoid follicles’ in oral amalgam-associated lichenoid lesions.. Oral Dis..

[28] Pawar R.R., Mattigatti S.S., Mahaparale R.R., Kamble A.P. (2016). Lichenoid reaction associated with silver amalgam restoration in a Bombay blood group patient: A case report.. J. Conserv. Dent..

